# Clinical comparison of liquid-based and conventional cytology of oral brush biopsies: a randomized controlled trial

**DOI:** 10.1186/s13005-018-0166-4

**Published:** 2018-05-29

**Authors:** Constanze Olms, Nathalie Hix, Heinrich Neumann, Maryam Yahiaoui-Doktor, Torsten W. Remmerbach

**Affiliations:** 1 Department of Dental Prosthodontics and Materials Science, University of Leipzig, Liebigstraße 12, 04103 Leipzig, Germany; 2 Department of Dental Prosthodontics and Materials Science, University of Leipzig, Liebigstraße 12, 04103 Leipzig, Germany; 3 Institute of Cytopathology, Am Propsthof 3, 53121 Bonn, Germany; 4 Institute for Medical Informatics, Statistics and Epidemiology (IMISE), University of Leipzig, Leipzig, Germany; 5 Section of Oral Medicine, Department of Head Medicine and Oral Health, University of Leipzig, Liebigstraße 10-14, 04103 Leipzig, Germany

**Keywords:** Collection methods, Preparation methods, Oral brush biopsy, Liquid-based cytology, Conventional cytology

## Abstract

**Background:**

Exfoliative cytology performed on oral brush samples can help dentists to decide, whether a given oral lesion is (pre-) malignant. The use of non-invasive brush biopsies as an auxiliary tool in the diagnosis of oral mucosal lesions has gained renewed interest since improvements in cytological techniques such as the development of adjuvant diagnostic tools and liquid-based cell preparation techniques.

**Methods:**

The aim of this study was to compare the quality of two different preparation techniques (cell collectors): the conventional transfer procedure to glass slides and the so-called liquid-based cytology preparation method. Cell smears were collected from 10 orally healthy individuals (mean age: 24 years) from the palatine mucosa at two different times (baseline and 4 weeks later). Slides of both techniques were stained by Giemsa (*n* = 40) and May-Gruenwald Giemsa (*n* = 40). The statistical analysis was performed with Excel.

**Results:**

On specimen analysis, the liquid-based cytology showed statistically significant improvement compared to conventional glass sides (*p* < 0.001). Thin layers, which were performed by liquid-based cytology showed significantly better results in the parameters (*p* < 0.001): uniform distribution, cellular overlapping, cellular disformation, mucus, microbial colonies and debris. The conventional glass slides approach showed more cell overlapping and contamination with extraneous material than thin layers, which were performed by Orcellex® Brush cell collectors.

**Conclusions:**

Both techniques are diagnostically reliable. The liquid-based method showed an overall improvement on sample preservation, specimen adequacy, visualization of cell morphology and reproducibility.

Liquid-based cytology simplifies cell collection due to easier handling and less transfer errors by dentists.

## Background

Oral brush biopsies were routinely used for cytological examination of the oral mucosa in the last decade [1–4]. The main advantages of oral brush cytology are: simple handling, well-tolerated, minimally invasive, and relatively painless for patient [5, 6]. The use of non-invasive brush biopsies as an auxiliary tool in the diagnosis of oral mucosal lesions has gained renewed interest since improvements in cytological techniques such as the development of adjuvant diagnostic tools and liquid-based cell preparation techniques [7–12]. Liquid-based cytology was originally developed to provide a near-monolayer of superficial cervicals cells by means of specimen filtration [13]. Results from uterine cervix examinations have shown that the liquid-based preparations reduce problems related to sampling error, poor transfer of the harvested cells by clinicians and fixation of cellular samples [7, 14–17]. The liquid-based cytology allows immediate fixation of cells while removing unwanted harvested material e.g. blood cells, mucus and debris. This preparatory technique produces so-called thin-layers, showing a clear background with evenly dispersed cells and producing more homogenous samples than conventional smears. Liquid-based techniques also reduce the proportion of unsatisfactory samples, thereby diminishing false negative results [7, 14–16]. The technique employs mucosal cell collection by plastic devices, which are then placed in a vial containing preservative liquid before being transported to the laboratory where collected liquid-based cytology samples are processed to produce slide preparations [6]. This is unlike conventional preparation in which exfoliated cells are spread onto a glass slide for cytological evaluation.

Sampling was carried out using cell collectors specially developed for the oral cavity [1, 18]. The requirements requested on cell collectors for oral smears are an optimal size and shape to provide access to the various areas of the oral cavity [2]. Cells of the superficial layers as well as middle and deep cell groups of the epithelium can be obtained with the collector. The preparations usually contain superficial as well as intermediate cells, basal cells occur only in isolated cases [1, 19–21]. The literature represent a number of brushes have been utilized for collecting oral mucosal cell. The review from Alsarraf et al. shows that the most commonly used brush is the cytobrush followed by the OralCDx® brush [6]. The most recent of all assessed brushes is the new custom-designed Orcellex® brush for use in the oral cavity [22].

The combination of a special plastic brush (Orcellex®) using liquid-based cytology shows current a potential method for early detection of oral cancer and precancer [23].

In the literature heterogeneous sampling techniques and brush cytology tools have been utilized for oral mucosal sampling. There are only a few comparative studies, which compare the conventional cytology with the liquid-based cytology for oral mucosa samples [7, 24].

Exfoliative cytology performed on oral brush samples can help dentists and other physicians to decide, whether a given oral lesion is (pre-) malignant or at an early and thus curable stage [2, 25, 26].

The aim of this prospective randomized study was to compare liquid-based and conventional cytology of oral brush biopsies in a standardized clinical experimental setting.

## Methods

This study was endorsed by the Leipzig Ethics Committee (file: 030–14-27,012,014). The study corresponds to the Consort guidelines [27]. In this study, the Helsinki Declaration was considered [28]. Ten test persons were included in the prospective randomized clinical study (female *n* = 3, male *n* = 7, mean age 24 years). The inclusion criteria were: age from 20 to 30 years, healthy, no mucosa lesions.

**Fig. 1:**
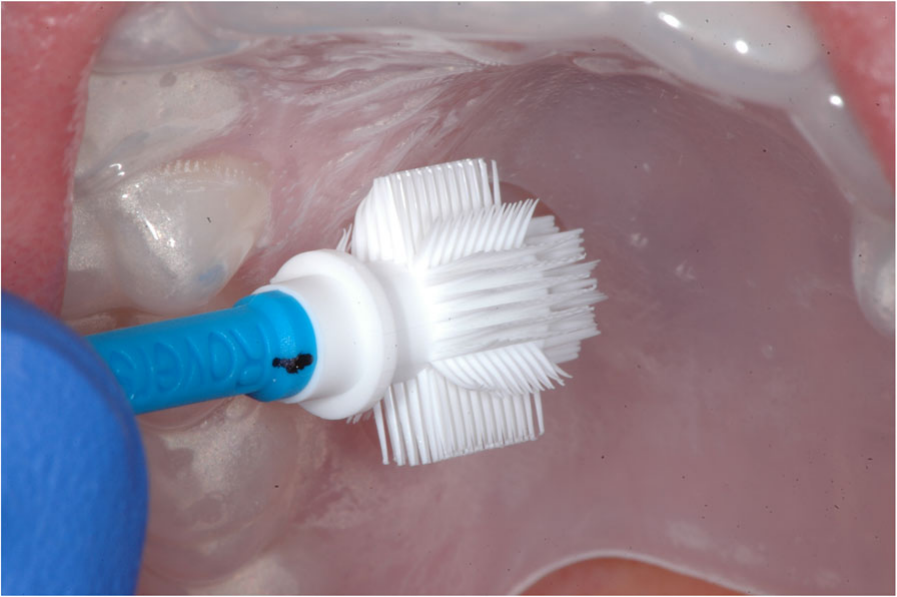
Splint for upper jaw with Orcellex® brush in situ

**Fig. 2:**
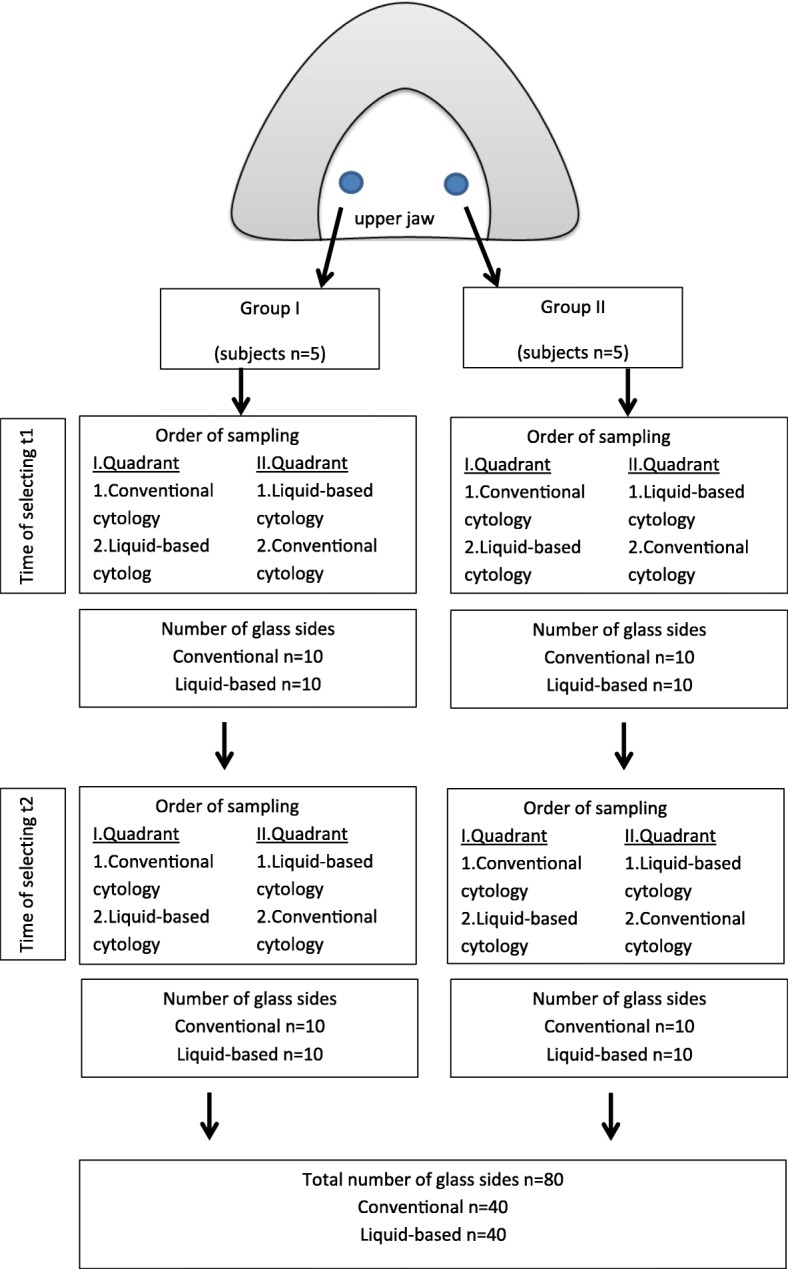
Flowchart

Two areas of the mucosa were selected on the palate of each test person. For the smear sampling of the palatal mucosa site, an individual splint was required for each test person to precisely locate the position during the sampling and to ensure the same smear areal. All splints were produced with a thermoforming machine (Vacfomat U, Dreve Dentamid GmbH, Unna, Germany) according to the manufacturer’s guidelines. The splint was inserted into the upper jaw of the test person to sign the exact position. The notch was chosen as small as possible to simulate a minimally invasive cell smear sampling, just so large that the cell collectors passed through. Afterwards, the smear sampling could be carried out with the aid of this fenestrated splint (Fig. 1). Two different cell collectors (cell smear sponge and cell smear brush) were used in this study. The cell sampling was randomized (Fig. 2). Two smears were taken at a certain time interval from the selected site of the palatal mucosa, namely, the first smear (t1), and then four weeks later (t2). In addition, two different cell preparation methods (conventional as well as liquid-based) were carried out in order to draw a comparison between the respective methods. Moreover, the smear preparations were stained by Giemsa and May-Gruenwald-Giemsa.

The conventional collection method, PapCone® Brushes (Otto Bock PUR LifeScience GmbH, Duderstadt, Germany) were used to obtain a cell smear from both quadrants. In order to perform the brush biopsies, the test person’s tongue was held by the assistant with a dental mirror, and the cell collecting was performed by the investigator on both sides at the corresponding mucosal areas. During the obtainment, it was necessary to ensure uniform contact between all bristles of the cell collector and the oral mucosa at the notch in the splint [29, 30]. The cell collector was rotated ten times about its own axis under moderate pressure on the mucosa [18, 31]. Subsequently the collected cells were transferred directly onto glass slides (Thermo Scientific™ SuperFrost©, Gerhard Menzel B.V. & Co. KG, Braunschweig, Germany) by rotating the sponge on it. For a homogeneous cell smear, in which the cells lie in one layer, the uniform rolling motion is decisive [32–34]. The slides were air-dried. In the laboratory the fixation was carried out by placing the glass slides in alcohol containing glass cuvettes for 20 min (methanol 99.8 vol.%, Th. Geyer GmbH & Co. KG, Renningen, Germany). After drying, Giemsa’s azur-eosin-methylene blue solution (Merck KGaA, Darmstadt, Germany) was used for staining the glass slides in according to the manufacturer guidelines. After further air-drying the glass slides were covered with Entellan (Merck, Darmstadt, Germany) and coverslips. They were stored at room temperature until evaluation.

For liquid-based cytology the cell collection method was carried out using Orcellex® Brushes (Rovers Medical Devices B.V., Oss, The Netherlands) according to the procedure described above.

One brush per sampling site was used to obtain a representative amount of epithelial cells. Following the brush biopsies by Orcellex® Brushes, fixation was carried out by separating the brush head from the applicator and placing it into the fixation liquid in the BD SurePath™ Collection Vial (BD Diagnostics - TriPath, Erembodegem, Belgium) [31]. In the laboratory of the Institute of Pathology (Mathias-Hospital, Rheine, Germany) the preparation of thin-layers (or monolayers) was done by the preparation procedure for SurePath™ preparations, which was originally developed for cervical smears [35]. Thin layers with a so-called settling chamber with a diameter of 13 mm were produced according to the manufacturer guidelines [19, 35]. The automatic staining of the preparations was carried out using the staining solutions (May-Gruenwald’s eosin-methylene blue solution and Giemsa’s azur-eosin-methylene blue solution, both Merck KGaA, Darmstadt, Germany) according to the manufacturer’s guidelines. Afterwards, the glass slides were covered and stored at room temperature until evaluation. All slides were examined by the same operator [36]. For comparative analysis of both techniques seven parameters were used: distribution, cellular overlapping, cellular disformation, mucus, blood cells, microbial colonies, debris. The categories were analyzed with “yes” (1) or “no” (0), and sum scores were calculated.

The individual scores for each of the seven parameters and the sum scores were tested for equality of variance using the F-Test. In the next step, the T-test was used to determine statistical differences between the two techniques. Significance was set at *p* < 0.05.

## Results

This study focuses on the cytological results of the two cell collectors and cell preparation methods. The probands of this study were oral healthy. No pathological cell deformations were observed.

A total of 80 glass slides were included in the evaluation. Of these 40 were produced with conventional cytology and 40 with the liquid-based preparation method. The staining of the 40 glass slides produced by conventional cytology was performed with the Giemsa staining solution. Of the 40 glass slides produced with liquid-based cytology (May-Gruenwald-Giemsa). The compared parameters are shown in Table 1.

**Table 1:** Comparison of different staining methods

staining method	Giemsa conventional	May-Gruenwald-Giemsa modified
used solution	Giemsa’s azur-eosin-methylene blue solution	May-Gruenwald’s eosin-methylene blue solution modified, Giemsa’s azur-eosin-methylene blue solution
staining cell nucleus	bluish	reddish - violet
staining cytoplasm	reddish	from bluish to reddish - violet
staining mucus	light purple	bluish
staining microbial colonies	Pink to reddish	reddish to purple
staining artifacts	blue to purple	blue to pink
staining background	light pink	clear

The smears were performed by two different cell collectors. The glass slides made with the PapCone® Brushes differ significantly from the smears taken with the Orcellex® Brush.

The Table 2 shows the results of the semi-quantitative analysis. The liquid-based preparations showed a statistically better (*p* < 0.001) uniform distribution, cellular overlapping, cellular disformation, mucus, microbial colonies and debris compared with those of conventional cytology (Table 2). No blood cells were identified on the thin layers. Only in two cases blood cells were detected on conventional glass slides. The sum score for liquid-based cytology was lower than the conventional cytology. These values are significant (*p* < 0.001).

**Table 2:** Sum score and significance

item	conventional cytology	liquid based cytology	T-test
nonuniform distribution	37	9	*p* < 0.001
cellular overlapping	38	17	*p* < 0.001
cellular disformation	35	17	*p* < 0.001
mucus	31	5	*p* < 0.001
blood cells	2	0	*p* = 0.160
microbial colonies	18	3	*p* < 0.001
debris	30	9	*p* < 0.001
Sum score	191/280	60/280	*p* < 0.001

**Fig. 3:**
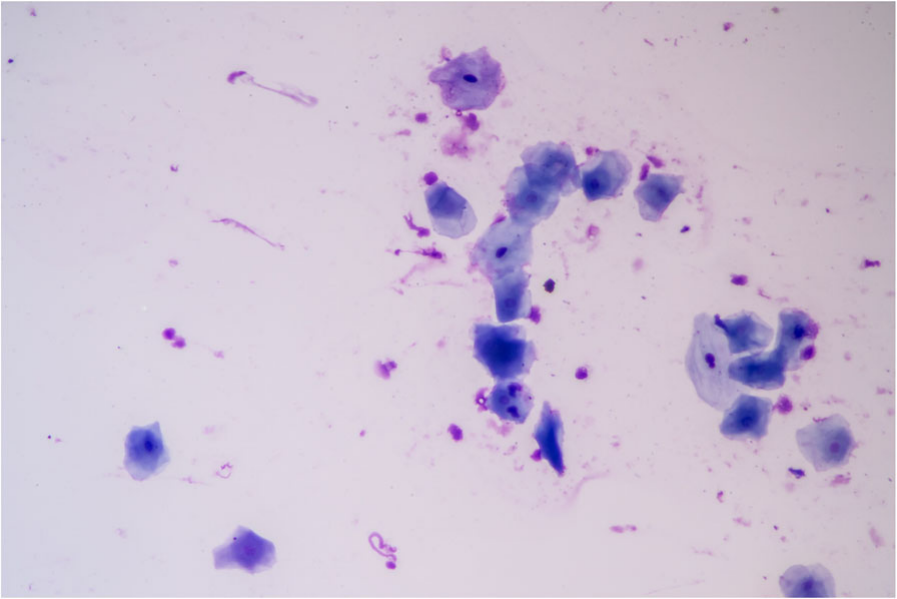
Conventional Giemsa (nonuniform distribution, cellular overlapping, mucus, debris)

The conventional preparations of the cell smear sponges varied in the amount of cells located thereon. Some glass slides were very full and contained lots of cells, other glass slides had fewer cells, and appeared much emptier. In addition, the distribution of the cells on the slides was uneven so that there were areas where the cells were clumped, but also areas where only few cells were present (see Fig. 3). Furthermore, it was noticeable that there were frequent overlaps, on the one hand, intersections with other cells, on the other hand also overlaps of the cells by themselves, for example by folding together the cells. In addition, some foreign material such as blood, mucus or food residues was found in these preparations.

The preparations which were produced with the liquid-based cell preparation method showed a uniform distribution of the cells in the circular evaluation area, even if there were glass slides with more and with fewer cells present. In addition, superpositions of the cells with other cells or with themselves occurred very rarely. There was hardly any foreign material compared to the smears with the PapCone® Brushes.

## Discussion

Results from this study have shown that the liquid-based cytology can offer significant advantages over the conventional exfoliative cytology in clinical practice.

First, some aspects of the materials and methods used will be discussed. Cell smears were taken from the selected areas of the palatine mucosa at two different times (first smear and four weeks later). In order to be independent of the current state of the cell cycle this time interval was chosen because the cell turnover rate of the oral mucosa epithelium is about ten to twelve days [37].

The comparison of these two cell collectors is of interest, since so far there have been very few investigations on different cell collectors in the literature. Previous studies compared, for example, a Cytobrush brush with a dermatological curette [38], with a wooden tongue spatula [39, 40] or with a metal spatula [41]. In a study by Reboiras-López et al. three different sampling devices (Cytobrush, dermatological curette, OralCDx Brush) were used to perform liquid-based cytology [21]. The used cell collectors (Orcellex® Brush, PapCone® Brush) differed in shape, the head of the PapCone® Brush was conical, and the head of the Orcellex® Brush was roll-shaped, resulting in a different approach to cell removal. Another significant difference was the texture of the cell collector heads, as the sponge was soft and pliable but the brush was rather firm and resistant. These characteristics influenced the cells collected during smear sampling and the appearance of the preparations. While taking the smears it was important to ensure a uniform, moderate contact pressure during rotation of the cell collector [31]. In the case of disregard, this could lead, for example, to uneven removal of mucosal cells. In addition, smear sampling was always carried out by the same investigator, because there can be individual differences between various practitioners, although there are guidelines for correct smear sampling. Comparing the smears of the two used cell collectors with regard to their cytological appearance, the following observations can be summarized:

On the one hand, the PapCone® Brush was used in combination with conventional cell preparation. An advantage of this method was the rapid processing of the preparations in the laboratory of the section of Clinical and Experimental Oral Medicine after smear sampling by the direct rolling out of the sponges on the glass slides and the following air drying, fixation and staining. The glass slides produced by the conventional preparation can be examined immediately [19]. Furthermore, the smear sampling with the softer texture of the spongy PapCone® Brush was more comfortable for the test person compared to the Orcellex® Brush. A disadvantage of the conventional exfoliative cytology was that irregular removal of the cell material was caused by the rolling-off process of the cell collector on the glass slides. By rolling-off the cell collector the cells were often concentrated in a line, while other areas were almost empty. In the areas with a lot of cell material, there were lots of cell overlapping, both overlaps of the cells by themselves and intersections with other cells. A further disadvantage of this conventional preparation technique was that, after rolling-off the collector, a considerable number of epithelial cells remained on the cell collectors. These remaining cells were discarded and thus were not included in the evaluation [19, 31]. Furthermore, some pollutant material, e.g. mucus, debris and blood cells, was present in the preparations. Such overlapping artifacts made the assessment and evaluation of the preparations partly difficult [31].

On the other hand, smear sampling was carried out using Orcellex® Brushes (Rovers Medical Devices B.V., Oss, Netherlands). The harder bristles of the cell smear brushes were noticeable for the test persons when taking the smear. In order to obtain sufficient cell material, twisting the brush ten times [42]. After taking the smears, the brush heads were placed directly into the fixation liquid and washed out. For further processing, the Collection Vials (BD Diagnostics - TriPath, Erembodegem, Belgium) were sent to the laboratory of the Institute of Pathology (Mathias-Spital, Rheine), where the preparation of thin-layers was done by the procedure for SurePath™ preparations. A disadvantage of the liquid-based preparation method was the increased time required for packaging and delivery of the samples as well as the more elaborate processing for the liquid-based preparation in the laboratory. Thus, the liquid-based preparation of the smears was technically more complex, but showed significant benefits over conventional cytology. The advantages of the monolayer technique were that they did not have to be processed immediately. After placing the brush heads in the fixation liquid, storage of more than 24 h is possible. Another positive feature of this method is that the sampled cells are washed out of the brush by the liquid and are in the solution. Thus, it can be assumed that by washing out the cells from the cell collectors a significantly higher proportion of representative epithelial cells is present for the evaluation, since less important cell material is lost by liquid-based method than with conventional exfoliative cytology [7, 19, 31]. Failure sources due to incorrect transfer or fixation by the investigator can be minimized by the monolayer preparation technique [19]. When the liquid was applied to the glass slide, no mechanical deformation took place and the cells retained their original shape. Furthermore, this procedure also resulted in a much more even distribution of the cells on the slide and thus less cell overlapping was found [2, 7, 19, 20, 31]. Another important advantage of this method was that the unwanted harvested material, such as blood cells, debris and mucus, was almost completely filtered out before being transferred to the slides [2, 19, 20, 31]. Therefore, the preparations showed less impurities and the background appeared clearer [7, 19]. Thus, an evaluation of these preparations proved to be much simpler and more accurate, since the cells were homogeneously distributed and almost without overlaps. In conclusion, both the conventional exfoliative cytology and the liquid-based method provide reliable results. However, the liquid-based preparation method showed obvious advantages, because it represented the cell morphology better and, due to the aforementioned advantages, it was superior to the conventional cytology [7, 38, 43]. Thus the liquid-based cytology not only provided better results, but also cell material was available for further investigation [38, 43].

## Conclusion

In summary, qualitative and quantitative differences in the preparations were detected, depending on the cell collector used and the cell preparation method. There was a superiority of the Orcellex® Brush in combination with the liquid-based monolayer technique.
